# Supplementary material for: The Distribution Characteristics of a 19-bp Indel of the PLAG1 Gene in Chinese Cattle
Source: Animals (Basel). 2019 Dec 4;9(12):1082. doi: 10.3390/ani9121082 (PMC6940828; doi:10.3390/ani9121082)
Supplement: Supplementary file 1 [file animals-09-01082-s001.docx]

**Supplementary Materials:**

**Table S1. Distribution of 37 cattle breeds of China as well as Angus and Holstein populations.**

| **Groups** | **Breeds (codes)** | **Sample size** | **Locality** | **North latitude (° N)** |
| --- | --- | --- | --- | --- |
|  |  |  |  |  |
| Northern group | Kazakh (KZ) | 24 | Yining,Yili, Xinjing, China | 46.2 |
|  | Yanbian (YB) | 26 | Yanbian, Jilin, China | 44.91 |
|  | Mongolian (MG) | 31 | Heshuo, Kuerl, Bayingolin, Xinjiang, China | 42.29 |
| Central group | Jiaxian Red (JX) | 24 | Jiaxian, Pingdingshan, Henan, China | 34.65 |
|  | Nanyang (NY) | 23 | Nanyang, Henan, China | 33.34 |
|  | Luxi (LX) | 27 | Jining, Shandong, China | 37.09 |
|  | Bohai Black (BH) | 16 | Binzhou, Shandong, China | 38.95 |
|  | Jinnan (JN) | 24 | Yuncheng, Shanxi, China | 35.42 |
|  | Qinchuan (QC) | 30 | Yangling, Shaanxi, China | 34.51 |
|  | Zaosheng(ZS) | 36 | Qingyang,Gansu,China | 36.03 |
| Southern group | Ji'an (JA) | 52 | Ji’an, Jiangxi, China | 27.12 |
|  | Jinjiang (JJ) | 12 | Gaoan, Yichuan, Jiangxi, China | 28.5 |
|  | Wannan (WN) | 24 | Jixi, Anhui, China | 31.95 |
|  | Weining (GZWN) | 32 | Weining, Bijie, Guizhou, China | 27.74 |
|  | Zaobei (ZB) | 11 | Zaoyang, Xiangyang, Hubei, China | 32.13 |
|  | Dabieshan (DBS) | 23 | Anqing, Anhui, China | 30.53 |
|  | Bashan (BS) | 32 | Xuanhan, Dazhou, Sichuan, China | 31.36 |
|  | Wenshan (WS) | 19 | Wenshan, Yunan, China | 23.41 |
|  | Dianzhong (DZ) | 24 | Chuxiong, Yunan, China | 25.04 |
|  | Guangfeng (GF) | 23 | Guangfeng, Shangrao, Jiangxi, China | 28.64 |
|  | Sanjiang (SJ) | 30 | Sanjiang, Baishi, Sichuan, China | 31.49 |
|  | Guanling (GL) | 28 | Guanling, Guizhou, China | 25.97 |
|  | Wuchuan (WC) | 23 | Fenggang, Guizhou, China | 28.61 |
|  | Shigatse Humped (SH) | 29 | Dingjie,Xizang, China | 28.57 |
|  | Diqing(DQ) | 24 | Diqing,Yunnan,China | 27.83 |
|  | Jiangcheng(JC) | 24 | Pu'er,Yunnan,China | 22.58 |
|  | Hainan(HN) | 23 | Haikou,Hainan,China | 20.02 |
|  | Dehong(DH) | 19 | Dehong,Yunnan,China | 24.43 |
|  | Weizhou(WZ) | 23 | Beihai,Guangxi,China | 21.49 |
|  | Sinan(SN) | 24 | Sinan,Guizhou,China | 27.94 |
|  | Zhaotong(ZT) | 31 | Zhaotong,Yunnan,China | 26.34 |
|  | Nandan(ND) | 25 | Nandan,Guangxi,China | 24.42 |
| Commercial group | Yunling(YL) | 451 | Kunming,Yunnan,China | 24.23 |
|  | Xianan(XN) | 12 | Miyang,Henan,China | 32.72 |
| Control group | Holstein(Holstein) | 20 | Yangling,Shanxi,China | 34.51 |
|  | Angus (Augus) | 28 | Yangling, Shaanxi, China | 34.51 |
| particular group | Tibetan(TB) | 27 | Changdu,Xizang,China | 29 |
|  | Total | 1354 |  |  |

**Table S2.** Genotypic and allele frequencies of the *PLAG1* gene across 37 cattle breeds.

| **Group** | **Breed** | **Genotype** | | | **Samples** | **Genotype frequencies** | | | **Allelic frequencies** | | **Average body height（cm)** | |
| --- | --- | --- | --- | --- | --- | --- | --- | --- | --- | --- | --- | --- |
|  |  | **WW** | **WD** | **DD** | **N** | **P-WW** | **P-WD** | **P-DD** | **PW** | **PD** | Male | Female |
| Northern group | Kazakh (KZ) | 19 | 1 | 4 | 24 | 0.7917 | 0.0417 | 0.1667 | 0.8125 | 0.1875 | 122.3±1.7 | 115.2±5.6 |
|  | Yanbian (YB) | 10 | 9 | 7 | 26 | 0.3846 | 0.3462 | 0.2692 | 0.5577 | 0.4423 | 131.5 | 122.5 |
|  | Mongolian (MG) | 26 | 0 | 5 | 31 | 0.8387 | 0.0000 | 0.1613 | 0.8387 | 0.1613 | 119.7 | 113.6±3.4 |
| Central group | Jiaxian Red (JX) | 2 | 1 | 21 | 24 | 0.0833 | 0.0417 | 0.8750 | 0.1042 | 0.8958 | 126.1±7.0 | 121.2±6.2 |
|  | Nanyang (NY) | 1 | 1 | 21 | 23 | 0.0435 | 0.0435 | 0.9130 | 0.0652 | 0.9348 | 144.9±9.6 | 126.3±7.1 |
|  | Luxi (LX) | 1 | 10 | 16 | 27 | 0.0370 | 0.3704 | 0.5926 | 0.2222 | 0.7778 | 146.3±6.9 | 123.6±5.6 |
|  | Bohai Black (BH) | 9 | 0 | 7 | 16 | 0.5625 | 0.0000 | 0.4375 | 0.5625 | 0.4375 | 129.6±4.0 | 116.6±4.9 |
|  | Jinnan (JN) | 6 | 1 | 17 | 24 | 0.2500 | 0.0417 | 0.7083 | 0.2708 | 0.7292 | 138.6 | 117.4 |
|  | Qinchuan (QC) | 1 | 1 | 28 | 30 | 0.0333 | 0.0333 | 0.9333 | 0.0500 | 0.9500 | 141.7±13.9 | 127.2±5.8 |
|  | Zaosheng(ZS) | 6 | 8 | 22 | 36 | 0.1667 | 0.2222 | 0.6111 | 0.2778 | 0.7222 | 137.7±6.3 | 126.8±4.6 |
| Southern group | Ji'an (JA) | 0 | 46 | 6 | 52 | 0.0000 | 0.8846 | 0.1154 | 0.4423 | 0.5577 | 114.50±6.43 | 105.48±7.78 |
|  | Jinjiang (JJ) | 1 | 5 | 6 | 12 | 0.0833 | 0.4167 | 0.5000 | 0.2917 | 0.7083 | 121.66±6.49 | 107.31±5.15 |
|  | Wannan (WN) | 2 | 14 | 8 | 24 | 0.0833 | 0.5833 | 0.3333 | 0.3750 | 0.6250 | 120.62±4.51 | 112.08±2.21 |
|  | Weining (GZWN) | 2 | 2 | 28 | 32 | 0.0625 | 0.0625 | 0.8750 | 0.0938 | 0.9063 | 113.8±7.4 | 108.6±5.0 |
|  | Zaobei (ZB) | 1 | 7 | 3 | 11 | 0.0909 | 0.6364 | 0.2727 | 0.4091 | 0.5909 | 126.6±0.7 | 115.2±0.9 |
|  | Dabieshan (DBS) | 2 | 16 | 5 | 23 | 0.0870 | 0.6957 | 0.2174 | 0.4348 | 0.5652 | 119.4 | 110.7 |
|  | Bashan (BS) | 6 | 9 | 17 | 32 | 0.1875 | 0.2813 | 0.5313 | 0.3281 | 0.6719 | 124±5.8 | 114±4.4 |
|  | Wenshan (WS) | 2 | 10 | 7 | 19 | 0.1053 | 0.5263 | 0.3684 | 0.3684 | 0.6316 | 120.1±5.9 | 115.3±2.3 |
|  | Dianzhong (DZ) | 2 | 12 | 10 | 24 | 0.0833 | 0.5000 | 0.4167 | 0.3333 | 0.6667 | 105.0±6.9 | 101.9±5.2 |
|  | Guangfeng (GF) | 0 | 14 | 9 | 23 | 0.0000 | 0.6087 | 0.3913 | 0.3043 | 0.6957 | 130.36±9.07 | 110.85±4.62 |
|  | Sanjiang (SJ) | 2 | 12 | 16 | 30 | 0.0667 | 0.4000 | 0.5333 | 0.2667 | 0.7333 | 116.0±6.4 | 111.0±5.7 |
|  | Guanling (GL) | 4 | 4 | 20 | 28 | 0.1429 | 0.1429 | 0.7143 | 0.2143 | 0.7857 | 120.4±5.8 | 112.9±4.2 |
|  | Wuchuan (WC) | 1 | 16 | 6 | 23 | 0.0435 | 0.6957 | 0.2609 | 0.3913 | 0.6087 | 120.6±10.8 | 117.6±8.6 |
|  | Shigatse Humped (SH) | 11 | 11 | 7 | 29 | 0.3793 | 0.3793 | 0.2414 | 0.5690 | 0.4310 | 131.9±5.1 | 128.9±4.7 |
|  | Diqing(DQ) | 10 | 7 | 7 | 24 | 0.4167 | 0.2917 | 0.2917 | 0.5625 | 0.4375 | 106.3 | 105 |
|  | Jiangcheng(JC) | 1 | 15 | 8 | 24 | 0.0417 | 0.6250 | 0.3333 | 0.3542 | 0.6458 |  |  |
|  | Hainan(HN) | 1 | 12 | 10 | 23 | 0.0435 | 0.5217 | 0.4348 | 0.3043 | 0.6957 |  |  |
|  | Dehong(DH) | 0 | 12 | 7 | 19 | 0.0000 | 0.6316 | 0.3684 | 0.3158 | 0.6842 |  |  |
|  | Weizhou(WZ) | 0 | 20 | 3 | 23 | 0.0000 | 0.8696 | 0.1304 | 0.4348 | 0.5652 | 112.1±6.5 | 104.3±4.3 |
|  | Sinan(SN) | 8 | 9 | 7 | 24 | 0.3333 | 0.3750 | 0.2917 | 0.5208 | 0.4792 |  |  |
|  | Zhaotong(ZT) | 7 | 9 | 15 | 31 | 0.2258 | 0.2903 | 0.4839 | 0.3710 | 0.6290 | 116.5±5.7 | 110.2±6.6 |
|  | Nandan(ND) | 1 | 16 | 8 | 25 | 0.0400 | 0.6400 | 0.3200 | 0.3600 | 0.6400 | 109.3±6.5 | 104.9±5.1 |
| Commercial group | Yunling(YL) | 154 | 74 | 223 | 451 | 0.3415 | 0.1641 | 0.4945 | 0.4235 | 0.5765 |  |  |
|  | Xianan(XN) | 4 | 0 | 8 | 12 | 0.3333 | 0.0000 | 0.6667 | 0.3333 | 0.6667 | 142.5±8.5 | 135.5±9.2 |
| Control group | Holstein(Holstein) | 19 | 9 | 0 | 28 | 0.6786 | 0.3214 | 0.0000 | 0.8393 | 0.1607 | 155~175 | 135~155 |
|  | Angus (Augus) | 7 | 1 | 12 | 20 | 0.3500 | 0.0500 | 0.6000 | 0.3750 | 0.6250 | 130 | 118.9 |
| Total |  | 329 | 394 | 604 | 1327 | 0.2479 | 0.2969 | 0.4552 | 0.3964 | 0.6036 |  |  |
| Particular group | Tibetan(TB) | 27 | 0 | 0 | 27 | 1.0000 | 0.0000 | 0.0000 | 1.0000 | 0.0000 | 104 | 99.9 |
